# Identification of Personality-Related Candidate Genes in Thoroughbred Racehorses Using a Bioinformatics-Based Approach Involving Functionally Annotated Human Genes

**DOI:** 10.3390/ani13040769

**Published:** 2023-02-20

**Authors:** Tamu Yokomori, Aoi Ohnuma, Teruaki Tozaki, Takao Segawa, Takuya Itou

**Affiliations:** 1 Nihon University Veterinary Research Center, Fujisawa 252-0880, Kanagawa, Japan; 2 Genetic Analysis Department, Laboratory of Racing Chemistry, Utsunomiya 320-0851, Tochigi, Japan

**Keywords:** horse, Thoroughbred, personality, genetics, polymorphism, orthologous, bioinformatics

## Abstract

**Simple Summary:**

By using the Thoroughbred genome variant database to identify orthologues to personality-related human genes, we identified 18 potential personality-related genes in horses. These candidate genes have a total of 55 variants that cause amino acid substitutions when compared to the EquCab3.0 reference genome that may impact the function of the proteins encoded by these genes. Moreover, 15 of the 18 genes have not previously been linked to personality in horses, suggesting that this exploratory approach of related genes using human evidence can be useful for equine behavioral genetics. Although using this bioinformatics approach is less useful for investigating genes affecting personality in horses than it is in humans due to a lack of supporting personality research, this study highlights the potential for the identification of candidate genes. If future studies with equine behavioral datasets validate these potential personality–gene associations, this bioinformatics strategy may become important in the field of equine genetics.

**Abstract:**

Considering the personality traits of racehorses (e.g., flightiness, anxiety, and affability) is considered essential to improve training efficiency and decrease accident frequency, especially when retraining for a second career that may involve contact with inexperienced personnel after retiring from racing. Studies on human personality-related genes are frequently conducted; however, such studies are rare in horses because a consistent methodology for personality evaluation is lacking. Using the recently published whole genome variant database of 101 Thoroughbred horses, we compared horse genes orthologous to human genes related to the Big Five personality traits, and identified 18 personality-related candidate genes in horses. These genes include 55 variants that involve non-synonymous substitutions that highly impact the encoded protein. Moreover, we evaluated the allele frequencies and functional impact on the proteins in terms of the difference in molecular weights and hydrophobicity levels between reference and altered amino acids. We identified 15 newly discovered genes that may affect equine personality, but their associations with personality are still unclear. Although more studies are required to compare genetic and behavioral information to validate this approach, it may be useful under limited conditions for personality evaluation.

## 1. Introduction

In the modern world, horses are used in various sports disciplines such as racing, show jumping, and dressage, as well as in various equine-assisted therapeutic and educational programs. Thoroughbred horses are mainly used in racing, but they have limited use after their retirement. In terms of animal welfare, international bodies such as the International Forum for the Aftercare of Racehorses (IFAR) have undertaken efforts to retrain racehorses and develop their post-retirement careers [1,2,3]. Personality and temperament are critical factors when selecting an appropriate individual for a specific purpose [4]. Since the learning ability of horses differs based on their personality and temperament, comprehensive knowledge of these aspects is crucial to improving the success of retraining [5,6]. Notably, no studies have investigated how temperament or personality influences performance or training.

Failures in the post-retirement transition from horseracing often occur in careers that involve greater contact with strangers or inexperienced personnel. One major problem for such horses is increased accident rates with inexperienced riders [7,8]. In other words, appropriate retraining is not conducted to capacitate the horse for amateurs. Therefore, carefully selecting horses for specific careers and setting a retraining direction in accordance with their intended use in their second career is expected to increase the rate of successful retraining.

Recently, twin or family research and whole-genome association studies have revealed human personality and related diseases to be partially controlled by genes [9,10,11]. The personality of horses may similarly be influenced by genetic factors, and additively by environmental factors [12,13]. For example, the equine *ASIP* genotype that influences coat color is associated with a self-reliant temperament [14], and a single nucleotide polymorphism (SNP) in the variable number of tandem repeats region of the equine DRD4 gene is significantly associated with curiosity and vigilance [15]. Moreover, the oxytocin and the dopaminergic pathways are associated with anxiety or fearfulness in horses, which constitute a temperament called “Neuroticism” in humans; oxytocin is also related to trainability [16,17]. Nine genes have been proposed to be personality-related candidate genes in horses [18]; however, few studies on each personality-related gene have been reported [15,19]. A lack of adequate methods for conducting personality-related research in horses is the primary reason for limited progress in this field when compared to the extensive research conducted in humans [20]. If equine personality genetics advance, a genetic test could be used to help select an ideal second career for retired racehorses, thus enabling more specialized retraining.

In humans, multiple personality-related genes have been identified by genome-wide association studies and the candidate gene approach. Recently, whole genome sequencing of 101 Thoroughbreds was performed, and the whole genome variant database of this population was published [21]. Therefore, we aimed to identify horse genes that are orthologous with human personality-related genes and develop a method for identifying equine personality-related gene candidates by referring to the variants of these genes extracted from the whole genome variant database.

## 2. Materials and Methods

**Figure 1:**
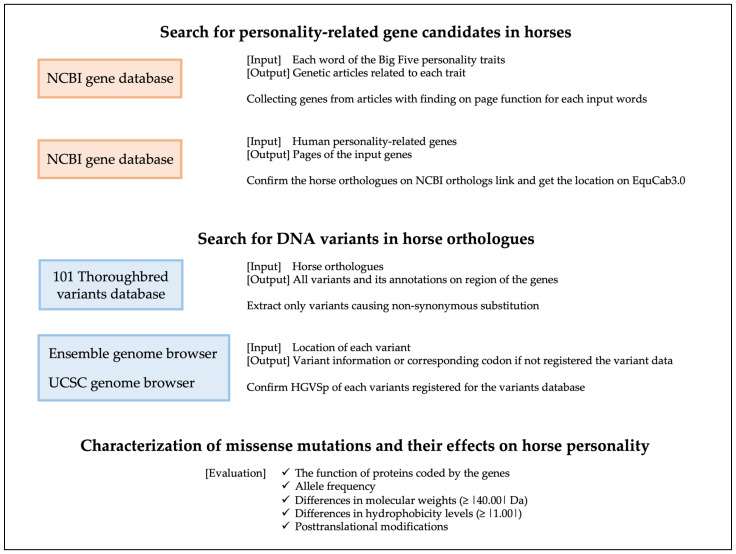
Methods pipeline. This shows the databases used in colored boxes, along with the inputs and outputs for each to the right side of each box. The sentences below the outputs show the objective for using each database. Finally, we list the items used to evaluate the importance of individual SNPs and their potential effect on personality.

In Figure 1, we show the referenced database, inputs and outputs, and our pipeline from methods to results.

### 2.1. Search for Personality-Related Candidate Genes in Horses

Firstly, human personality-related genes were identified using the search term “personality trait” in the National Center for Biotechnology Information (NCBI) gene database [22]. Subsequently, relevant articles dealing with the identified genes were collected; the articles discussed each facet of the Big Five personality traits: Agreeableness, Conscientiousness, Extraversion, Neuroticism, and Openness. These categories have been determined to be heritable in humans [23]. Horse genes orthologous to the identified human genes were searched against the NCBI gene database.

### 2.2. Search for DNA Variants in Equine Personality-Related Gene Candidates

The variants of each gene were sorted using a whole genome variant database of 101 Thoroughbreds that uses EquCab3.0 as a reference genome [21,24]. Variants causing non-synonymous substitution were selected as mutations likely to highly impact protein function. Variant information was checked using the Ensembl Genome Browser and the University of California Santa Cruz (UCSC) Genome Browser [25,26].

### 2.3. Characterization of Missense Mutations and Their Effects on Equine Personality

Based on variants’ codon mutations, the differences in molecular weights and hydrophobicity index levels of amino acids were manually calculated before and after substitutions. Furthermore, substitutions with a molecular weight difference ≥|40.00| Da and a hydrophobicity level ≥|1.00| were treated as highly significant [27]. Disulfide-bond and posttranslational modifications were investigated in regard to their effect on the conformation or function of encoded proteins.

## 3. Results

### 3.1. Search for Personality-Related Candidate Genes in Horses

Twenty-eight human personality-related genes were extracted from PubMed by searching the NCBI gene database. The horse orthologues for all 28 genes were found in the horse genome based on gene annotations obtained using Ensembl.

Among the 28 identified genes, *ANKK1*, *APOE*, *BDNF*, *CNR1*, *COMT*, *DRD4*, *IL6*, and *SLC6A4* were associated with two or more personality traits. *COMT* was specifically associated with all personality traits. Among all the traits explored, Neuroticism was associated with the largest number of genes (23 genes), and Extraversion, Conscientiousness, Agreeableness, and Openness were associated with six, four, three, and four genes, respectively.

### 3.2. Search for DNA Variants in Equine Personality-Related Gene Candidates

After searching all variants of the 28 orthologues in the Thoroughbred variant database, 55 variants causing non-synonymous substitution in 18 different genes were extracted (Table S1). Among the 55 variants, 54 were SNPs that caused a substitution of one nucleotide for another, and one was an insertion that caused the mutation of a nucleotide to a long sequence of nucleotides. These mutations in horses were not found to be in corresponding locations in the human genome, as checked using the Ensembl and UCSC genome browsers.

SNPs in the coding region of *ANKK1*, which is associated with Neuroticism and Extraversion, were the most frequent (13) among the identified variants. In contrast, nine out of the ten genes excluded from the candidate search, which have no variants with non-synonymous substitutions, were associated with Neuroticism. Out of the 18 identified genes, in 15 the substitutions were principally related to major signaling pathways in the central nervous system, such as the intracellular signaling pathway, neurotransmission pathway, the hypothalamic–pituitary–adrenal axis, and intercellular connections. Additionally, *APOE* and *BDNF*, the neurotrophic-related factors related to the accumulation of amyloid beta in the brain, and *PER3*, which is related to the regulation of circadian rhythm, were identified as personality-related genes (Table 1).

### 3.3. Characterization of Missense Mutations and Their Effects on Equine Personality

**Figure 2:**
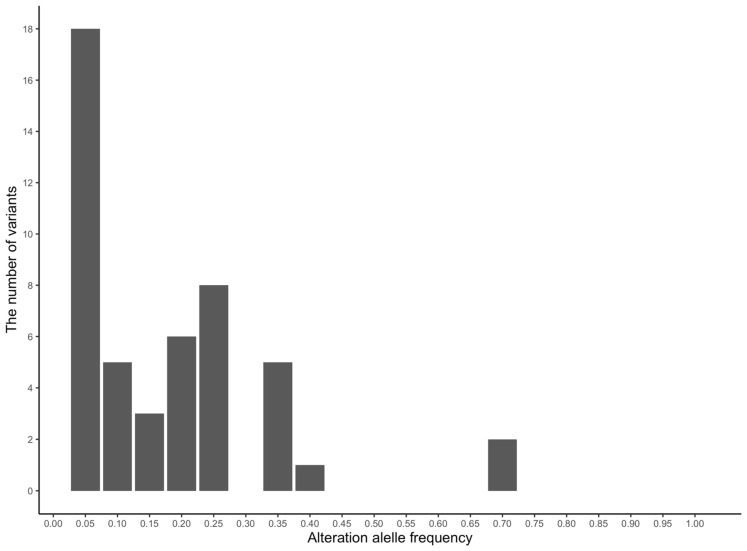
Distribution of alteration allele frequencies of the 18 most frequently identified horse personality-related genes. The histogram depicts the number of SNPs in each frequency hierarchy value. The total is 48 SNPs, and the maximum and minimum values of alteration allele frequency are 0.668 and 0.005, respectively.

Among the 55 identified variants, the 18 most frequent SNPs (37.50%) exhibited ≤ 0.05 minor allele frequency (MAF), whereas 0.3–0.7 alteration allele frequency was detected in 8 SNPs (16.67%). Notably, two SNPs located at chr11:44188160 and chr11:44188161 on *SLC6A4* both exhibited a high frequency of 0.668 (Table 2, Figure 2).

Codon insertion was not registered for the variant located at chrX:36800238 on *MAOA* in either the Ensembl or the UCSC genome database; however, codon deletion was recorded at the same location in the European Variation Achieve database. Moreover, allele frequency data for *MAOA*, which is on a sex chromosome, were not published in the Thoroughbred variant database.

Variants involving missense mutations were summarized as Human Genome Variant Society protein nomenclature (HGVSp) based on their amino acid location and substitution. Differences in molecular weight and hydrophobicity index owing to substitutions were analyzed for each variant (Table 3). Eight variants registered a molecular weight difference of ≥|40.00| Da, and fifteen variants resulted in ≥|1.00| deviation in the hydrophobicity index. Moreover, two HGVSp were identified for the variants at chr3:31382147 and chr3:31845727 on *CDH13*. The codon locations vary in each splice variant.

## 4. Discussion

In this study, 28 human personality-related genes and their orthologous genes in horses were identified. Among them, 18 genes were detected that have 55 DNA polymorphisms annotated as non-synonymous substitutions in the whole genome variant database of a population of 101 Thoroughbreds. Missense mutations potentially influence the structure of proteins, thereby altering their functions. Therefore, these 18 genes were putative candidate genes affecting personality diversity in horses. Moreover, among these genes, *BDNF*, *HTR2A*, and *MAOA* were reported as candidates principally associated with personality in horses [13]. Collectively, candidate genes can be identified using this bioinformatics method.

Amino acid substitution significantly alters molecular weight, alters the primary and secondary structure, and influences the functional characteristics of proteins [28]. Eight variants were detected that cause significant changes in the molecular weight of amino acids encoded by seven genes, namely, *ANKK1*, *APOE*, *CDH13*, *DGKH*, *FAAH*, *HSD11B1*, and *MAOA*. Accordingly, these variants could influence the protein–protein interaction network and, therefore, pathways associated with personality.

Altered hydrophobicity levels can disrupt the configuration of amino acids, resulting in changes in protein conformation and native folding [29]. Fifteen variants of seven genes—*ANKK1*, *APOE*, *CDH13*, *DRD2*, *FAAH*, *GABRA6*, and *MAOA*—were identified, which significantly impacted the hydrophobicity of the resulting amino acids. Therefore, the protein transportation and intercellular signaling pathways regulated by these genes may be affected by these amino acid alterations.

Gain or loss of an amino acid that participates in posttranslational modifications can alter the activity or localization of the protein, thereby affecting horse personality diversity. The variant at chrX:36799409 in *MAOA* removes cysteine, which is susceptible to S-nitrosylation; therefore, the enzyme activity and expression level of monoamine oxidase A (MAOA) may also be affected [30]. Variants causing gain or loss of asparagine, which is susceptible to N-glycosylation, were detected in genes encoding MAOA, ankyrin repeat and kinase domain containing I protein (ANKK1), and period circadian protein homolog 3 protein (PER3) [31]. Changes in folded protein structure influence the enzymatic activity of MAOA and ANKK1 and the efficiency of transportation and signal transduction of PER3. Additionally, serine, tyrosine, and threonine are frequently subjected to phosphorylation, which plays a role in cell signal switching [32]; variants causing gain or loss of these amino acids were detected in *MAOA*, *ANKK1*, *PER3*, *HSD11B1*, *NPY*, *CDH13*, *DGKH*, *GABRA6*, *P2RX7*, *COMT*, *HTR2A*, and *LEP*. Hence, amino-acid-specific posttranslational modification influences protein function and may also likely contribute to horse personality diversity.

Notably, many of the identified genes were related to Neuroticism. This personality trait indicates a neuropathological characteristic [33] and has been linked to a depression-related phenotype [34]. Moreover, *ANKK1* and *PER3* were associated with Extraversion, *NPY* was associated with Conscientiousness, *CDH13* and *CNR1* were associated with Agreeableness, and *DGKH* was associated with Openness; each of these genes was also associated with Neuroticism, suggesting that they mediate a trade-off or compatibility between the two personality traits.

Among the 55 variants, the fact that 37.50% of the SNPs represented 5% or less MAF may not relate to the universal diversity of personality in the Thoroughbred population because allele frequency of variants with low harmfulness to an organism tends to increase via genetic drift [35]. Accordingly, the widely expanded SNPs (MAF: 0.3–0.5) may be more useful for comprehending the universal diversity of Thoroughbred personality.

A substitution from cysteine to arginine at chrX:36799409 on *MAOA* may result in the loss of a disulfide bridge, which is the primary covalent protein bond. Consequently, this substitution may exacerbate the Neuroticism trait associated with *MAOA* since it causes the protein conformation to become unstable [36,37]. Changes at loci including chr3:31845727 on *CDH13*, chr7:22331004 on *ANKK1*, chr2:12174492 on *FAAH*, and chrX:36799409 on *MAOA*, can cause changes in both the configuration of amino acids and the conformation of coded proteins, and may thus functionally impact molecular weights and hydrophobicity levels. In particular, SNPs at chr3:31845727 and chrX:36799409 may cause alterations in protein activity due to the loss of serine and cysteine, respectively. These SNPs can thus potentially increase or decrease the output intensities of the corresponding personalities.

Although personality has been reported to vary between horse breeds, this can likely be attributed to environmental differences that are involved in managing horses and the variation of variant distribution in the population due to selective pressures on each breed [38]. Therefore, variants in personality-related candidate genes are assumed to be at the same loci and serve the same function in other breeds as in Thoroughbreds.

An escape response that occurs due to a frightening stimulus can lead to injuries when riding a horse; however, desensitization training can reduce this response [39,40]. Selectively retraining horses with low Neuroticism can improve the efficiency of converting racehorses for general horse riding. *MAOA*, which is related to Neuroticism in humans, may be responsible for the difference in escape response that exists between sexes [41]. Furthermore, horses’ reactivity to humans is considered to be related to the Openness trait, with a facet of curiosity [42]. Openness should be evaluated when retraining horses for use in careers that involve being touched by inexperienced and passive personnel because horses with high Openness may tend to demonstrate behaviors that have the potential to harm humans, such as biting hands or pulling clothes.

## 5. Conclusions

We developed a novel bioinformatics approach to identify equine personality-related candidate genes using related knowledge of human genes and the genome variant database of 101 Thoroughbreds. This strategy could support research progress in using genetics to predict equine personality, and it is expected to trigger investigations into the associated protein functions. Moreover, this findings may aid in expanding the knowledge of the possibility of selecting a second career for Thoroughbred horses based on genetics. This research can thus be used to improve the health and welfare of horses. As of now, this bioinformatics approach should be treated as preliminary since these gene–personality associations in horses have not been confirmed. Therefore, more in vivo studies and functional analyses are needed to demonstrate the accuracy and strength of these associations.

## Figures and Tables

**Table 1:** Categories of biological function and the classification of equine personality-related gene candidates.

Biological Function			Gene
Intracellular signaling pathway			*DGKH*, *FAAH*
Neurotransmission pathway	Monoamine neurotransmission	Dopaminergic signaling system	*ANKK1*, *DRD2*
		Serotonergic signaling system	*HTR2A*, *SLC6A4*
		Monoamine inactivation enzyme	*COMT*, *MAOA*
	Amino acid neurotransmission	GABAergic signaling system	*GABRA6*
		Endogenous cannabinoid signaling system	*CNR1*
	Peptide neurotransmission		*LEP*, *NPY*
	Purinergic signaling system		*P2RX7*
Hypothalamic–pituitary–adrenal axis			*HSD11B1*
Neurotrophic-related factor			*APOE*, *BDNF*
Intercellular connections			*CDH13*
Circadian rhythm			*PER3*

**Table 2:** Alleles and variant frequencies in the 18 most frequently identified equine personality-related genes.

Gene	Chromosome	Position	Reference Allele	Alteration Allele	Reference Allele Frequency	Alteration Allele Frequency
*FAAH*	2	12167952	G	A	0.995	0.005
		12174477	G	A	0.936	0.064
		12174492	G	T	0.955	0.045
*PER3*	2	43209639	A	G	0.683	0.317
		43231315	A	G	0.678	0.322
		43240108	C	A	0.822	0.178
*CDH13*	3	31079056	T	C	0.911	0.089
		31230472	G	A	0.955	0.045
		31382147	C	T	0.757	0.243
		31845727	C	T	0.995	0.005
*NPY*	4	55899514	C	G	0.861	0.139
*LEP*	4	83529565	C	T	0.891	0.109
*HSD11B1*	5	25786262	T	G	0.767	0.233
*ANKK1*	7	22319266	A	G	0.782	0.218
		22319267	T	A	0.787	0.213
		22319365	G	C	0.772	0.228
		22319396	T	C	0.995	0.005
		22319430	G	T	0.995	0.005
		22319446	A	G	0.782	0.218
		22319470	G	A	0.787	0.213
		22326586	A	G	0.698	0.302
		22327351	G	A	0.698	0.302
		22329824	C	T	0.975	0.025
		22330242	G	A	0.995	0.005
		22331004	C	A	0.960	0.040
		22331042	G	A	0.941	0.059
*DRD2*	7	22348064	T	A	0.995	0.005
*BDNF*	7	96310373	A	T	0.812	0.188
*COMT*	8	432351	T	C	0.842	0.158
		434481	A	G	0.886	0.114
*P2RX7*	8	24214858	A	G	0.644	0.356
*APOE*	10	15713778	A	G	0.995	0.005
		15714427	A	G	0.832	0.168
		15714824	T	G	0.995	0.005
*CNR1*	10	41805559	C	T	0.946	0.054
*SLC6A4*	11	44188160	A	G	0.332	0.668
		44188161	C	T	0.332	0.668
		44192165	T	C	0.985	0.015
		44200439	A	C	0.970	0.030
*GABRA6*	14	17329705	A	C	0.950	0.050
		17329709	C	T	0.842	0.158
		17329851	A	T	0.777	0.223
		17329886	T	C	0.985	0.015
		17338413	A	G	0.673	0.327
		17343960	C	T	0.926	0.074
*HTR2A*	17	23797786	A	G	0.990	0.010
*DGKH*	17	27918092	G	T	0.832	0.168
		28048492	A	G	0.985	0.015
*MAOA*	X	36799409	T	C	NA	NA
		36799613	G	A	NA	NA
		36800118	C	G	NA	NA
		36800211	T	C	NA	NA
		36800238	C	*	NA	NA
		36800403	G	T	NA	NA
		36801932	A	C	NA	NA

*: CATCTTTCCCGTTCTTGGGCTCCTCCCAAGTGTCGGTACTAGGGTCCGCCCCGCCATCAGTGCCAACTCCCC-CTGGAGTTCGGTACAAGG. Reference and alteration allele frequency: The values were extracted from the genome variant information database of 101 Thoroughbreds. The database uses EquCab3.0 as a reference sequence. NA: Not available. Data on allele frequencies of *MAOA* are lacking because this gene is on a sex chromosome, which is not published in the Thoroughbred variant database.

**Table 3:** Amino acid alterations and the differences in molecular weights and hydrophobicity levels caused by each variant.

Gene	Chromosome	Position	HGVSp	Annotation	Molecular Weight Difference	Hydrophobicity Index Difference
*FAAH*	2	12167952	A96V	non-synonymous coding	28.06	0.46
		12174477	A13V	non-synonymous coding	28.06	0.46
		12174492	A8D	non-synonymous coding	44.01	−1.52
*PER3*	2	43209639	L837S	non-synonymous coding	−26.08	−1.24
		43231315	V465A	non-synonymous coding	−28.06	−0.46
		43240108	K241N	non-synonymous coding	−14.07	0.72
*CDH13*	3	31079056	Y20H	non-synonymous coding	−26.04	−0.66
		31230472	V95I	non-synonymous coding	14.02	0.3
		31382147	R173W R134W	non-synonymous coding	30.03	3.34
		31845727	S696F S657F	non-synonymous coding	60.1	1.37
*NPY*	4	55899514	T139S	non-synonymous coding	−14.03	−0.13
*LEP*	4	83529565	T70M	splice site region non-synonymous coding	30.09	0.69
*HSD11B1*	5	25786262	Y88S	non-synonymous coding	−76.1	−0.44
*ANKK1*	7	22319266	I10V	non-synonymous coding	−14.02	−0.3
		22319267	I10N	non-synonymous coding	0.95	−2.16
		22319365	E43Q	non-synonymous coding	−0.98	1.22
		22319396	L53P	non-synonymous coding	−16.04	−0.94
		22319430	E64D	non-synonymous coding	−14.03	−0.16
		22319446	T70A	non-synonymous coding	−30.03	0.67
		22319470	E78K	non-synonymous coding	−0.94	−0.76
		22326586	I287V	non-synonymous coding	−14.02	−0.3
		22327351	R337H	non-synonymous coding	−19.05	2.13
		22329824	R411W	non-synonymous coding	30.03	3.34
		22330242	R550Q	non-synonymous coding	−28.05	3.01
		22331004	A804D	non-synonymous coding	44.01	−1.52
		22331042	E817K	non-synonymous coding	−0.94	−0.76
*DRD2*	7	22348064	K101I	non-synonymous coding	−15.02	2.88
*BDNF*	7	96310373	H14Q	non-synonymous coding	−9	0.88
*COMT*	8	432351	T231A	non-synonymous coding	−30.03	0.67
		434481	M159T	non-synonymous coding	−30.09	−0.69
*P2RX7*	8	24214858	S589G	non-synonymous coding	−30.02	0.66
*APOE*	10	15713778	Q17R	non-synonymous coding	28.05	−3.01
		15714427	M100V	non-synonymous coding	−32.06	0.44
		15714824	V232G	non-synonymous coding	−42.08	−0.6
*CNR1*	10	41805559	V263I	non-synonymous coding	14.02	0.3
*SLC6A4*	11	44188160	V539A	non-synonymous coding	−28.06	−0.46
		44188161	V539I	non-synonymous coding	14.02	0.3
		44192165	I408V	non-synonymous coding	−14.02	−0.3
		44200439	D36E	non-synonymous coding	14.03	0.16
*GABRA6*	14	17329705	I423M	non-synonymous coding	18.04	−0.74
		17329709	R422Q	non-synonymous coding	−28.05	3.01
		17329851	S375T	non-synonymous coding	14.03	0.13
		17329886	H363R	splice site region non-synonymous coding	19.05	−2.13
		17338413	V352A	non-synonymous coding	−28.06	−0.46
		17343960	E30K	non-synonymous coding	−0.94	−0.76
*HTR2A*	17	23797786	T39A	non-synonymous coding	−30.03	0.67
*DGKH*	17	27918092	S631Y	non-synonymous coding	76.1	0.44
		28048492	V12A	non-synonymous coding	−28.06	−0.46
*MAOA*	X	36799409	C24R	non-synonymous coding	53.04	−2.82
		36799613	G92S	non-synonymous coding	30.02	−0.66
		36800118	A142G A260G	non-synonymous coding	−14.02	−0.14
		36800211	L173P L291P	non-synonymous coding	−16.04	−0.94
		36800238	*	codon insertion	NA	NA
		36800403	W237L W355L	non-synonymous coding	−73.06	0.25
		36801932	N307T	non-synonymous coding	−13	0.73

*: S182SSFPFLGSSQVSVLGSAPPSVPSSPWSSVQG or S300SSFPFLGSSQVSVLGSAPPSVPSSPWSSVQG. HGVSp: Label of differences in the coded amino acid compared to that of reference sequence recommended by Human Genome Variation Society, showing reference amino acid, mutated position on the reference sequence, and altered amino acid. Molecular weight difference: Differences of amino acids caused by variants were calculated as (altered molecular weight) − (reference molecular weight). Values with molecular weight difference ≥|40.00| Da are underlined. The data corresponding to the variant X:36800238 in *MAOA* are lacking due to the long sequence insertion. Hydrophobicity index difference: This difference, caused by the amino acid substitution, was calculated as (altered hydrophobicity level) − (reference hydrophobicity level). Values with a difference ≥|1.00| are underlined. NA: Not available. Data are lacking corresponding to the insertion in *MAOA* due to the long sequence insertion.

## Data Availability

Data regarding human personality-related genes were collected via searches of the National Center for Biotechnology Information gene database [22]. Thoroughbred variant information was extracted from the Thoroughbred variant database [21]. Orthologous gene information and variant annotation were derived from the Ensembl Genome Browser [25] and UCSC Genome Browser [26].

## Supplementary Materials

The following supporting information can be downloaded at: https://www.mdpi.com/article/10.3390/ani13040769/s1, Table S1: Horse orthologous genes which have variants causing non-synonymous substitutions.
