# Supplementary material for: Identification of Personality-Related Candidate Genes in Thoroughbred Racehorses Using a Bioinformatics-Based Approach Involving Functionally Annotated Human Genes
Source: Animals (Basel). 2023 Feb 20;13(4):769. doi: 10.3390/ani13040769 (PMC9951868; doi:10.3390/ani13040769)
Supplement: Supplementary file 1 [file animals-13-00769-s001.zip › animals-2001284-supplementary.pdf]

Table S1. Horse orthologous genes which have variants causing non-synonymous substitutions

Gene ID	Full Name	Gene Symbol	Chromosome	Position
ENSECAG00000023711	fatty acid amide hydrolase	*FAAH*	2	12167952
				12174477
				12174492
ENSECAG00000018928	period circadian regulator 3	*PER3*	2	43209639
				43231315
				43240108
ENSECAG00000021162	cadherin 13	*CDH13*	3	31079056
				31230472
				31382147
				31845727
ENSECAG00000008726	neuropeptide Y	*NPY*	4	55899514
ENSECAG00000030283	leptin	*LEP*	4	83529565
ENSECAG00000019243	hydroxysteroid 11-beta dehydrogenase 1	*HSD11B1*	5	25786262
ENSECAG00000011781	ankyrin repeat and kinase domain containing 1	*ANKK1*	7	22319266
				22319267
				22319365
				22319396
				22319430
				22319446
				22319470
				22326586
				22327351
				22329824
				22330242
				22331004
				22331042
ENSECAG00000013567	dopamine receptor D2	*DRD2*	7	22348064
ENSECAG00000015952	brain derived neurotrophic factor	*BDNF*	7	96310373
ENSECAG00000005359	catechol-O-methyltransferase	*COMT*	8	434481
				432351
ENSECAG00000009949	purinergic receptor P2X 7	*P2RX7*	8	24214858
ENSECAG00000008236	apolipoprotein E	*APOE*	10	15713778
				15714427

				15714824
ENSECAG00000012581	cannabinoid receptor 1	*CNR1*	10	41805559
ENSECAG00000017112	solute carrier family 6 member 4	*SLC6A4*	11	44188160
				44188161
				44192165
				44200439
ENSECAG00000014012	gamma-aminobutyric acid type A receptor subunit alpha6	*GABRA6*	14	17329705
				17329709
				17329851
				17329886
				17338413
				17343960
ENSECAG00000024282	5-hydroxytryptamine receptor 2A	*HTR2A*	17	23797786
ENSECAG00000000237	diacylglycerol kinase eta	*DGKH*	17	27918092
				28048492
ENSECAG00000022277	monoamine oxidase A	*MAOA*	X	36799409
				36799613
				36800118
				36800211
				36800238
				36800403
				36801932

Gene ID: The identification ID used in Ensembl Genome Browser.
